# Unraveling the complex relationship between night shift work and diabetes: exploring mechanisms and potential interventions

**DOI:** 10.3389/fpubh.2025.1539679

**Published:** 2025-07-25

**Authors:** Yuye Zhu, Jing Mi

**Affiliations:** Xianyang Vocational Technical College, Xianyang, Shaanxi, China

**Keywords:** night shift work, type 2 diabetes mellitus, melatonin, circadian rhythms, sleep deprivation

## Abstract

It is a topic of increasing concern that the prevalence of night shift work in our 24/7 society is linked to diabetes mellitus. The purpose of this paper is to thoroughly examine existing research on the intricate connection between diabetes and night shift work, with a specific focus on underlying factors including insufficient sleep, disruptions in circadian rhythms, and changes in melatonin levels. Research has shown that the act of working overnight is linked to an increased likelihood of developing type 2 diabetes mellitus (T2DM), despite the fact that the exact reasons for this connection remain unclear. Insufficient sleep and disruption of the natural sleep-wake cycle, which are common consequences of working night shifts, can result in a reduced response to insulin and dysfunctional processing of glucose in the body. Moreover, changes in the production of melatonin, a hormone closely associated with the body's natural sleep-wake cycle, may have a significant impact. Although working the night shift presents numerous difficulties, interventions targeting the enhancement of sleep quality, adjustment of circadian rhythms, and increase in melatonin levels show potential in reducing the risk of developing T2DM. Further studies must persist in exploring these mechanisms and implementing successful tactics to safeguard the wellbeing of shift workers in our current societal context.

## 1 Introduction

In recent years, the workforce has undergone significant changes on a global scale, with a rising amount of people participating in non-traditional work arrangements like working during the night. The constantly evolving needs of a 24-h, 7-day-a-week society have sparked this change, impacting a wide range of sectors including healthcare, production, transportation, and customer service (1). Based on data from the Current Population Survey, about one-tenth of the labor force in Western countries is subject to evening employment, which encompasses fixed night shifts, varying shifts, and inconsistent work hours (2). Rotating employee schedules are crucial in keeping operations running smoothly as they guarantee a constant staffing of roles, thus preventing any disruptions in the workflow (3). Working at night is clearly defined as a period of work that spans for a minimum of seven consecutive hours, occurring between the hours of 12 o'clock in the morning and 5 o'clock in the early morning (4). The frequency and duration of shift schedules, as well as the consecutive workdays and rotation direction, can differ among companies (5).

Although shift work has certainly contributed crucial services and financial advantages, it has also sparked worries regarding its probable effects on the wellbeing of employees (6–11). Working during the night hours, which involves being exposed to man-made lighting, disturbs both the natural rhythm of social interaction and body functions, as well as the regularity of sleep (12–14). By utilizing these methods, there has been a suggestion that the chances of developing metabolic disorders may be increased, subsequently resulting in weight gain, obesity, breast cancer, heart disease, disruptions in metabolism, and potentially the development of type 2 diabetes mellitus (T2DM) (9, 12, 15–20). T2DM, a persistent and ongoing metabolic condition marked by hindrances in the regulation of glucose, is rapidly becoming a significant health issue on a worldwide scale (21). The International Diabetes Federation reported that around 463 million individuals across the globe had T2DM in 2019, and this figure is expected to reach 700 million by 2045. As the incidence of T2DM rises, it is crucial to grasp which elements of shift work schedules cause the most disruption and for whom (22). Having this understanding is crucial in creating specific plans for preventing diseases both in the early stages and later on. This could ultimately lessen the overall burden on society and reduce the financial impact associated with these diseases (23). Recent research indicates that working the night shift could potentially play a major role in the rising rates of T2DM. The body's circadian rhythm, known as its internal biological clock, controls a vast range of bodily processes, such as the cycles of sleeping and waking and the functioning of metabolism (24). The interruption of this natural body cycle caused by working night shifts can result in significant effects, such as changes in hormone release, eating patterns, and the body's ability to process insulin. Significant concerns have emerged, prompting further investigation into the potential correlation between working night shifts and an increased probability of developing T2DM (25, 26).

The primary objective of this literature review is to thoroughly examine and integrate previous studies in order to provide insight into the intricate connection between working night shifts and the susceptibility to T2DM. The topic will thoroughly investigate the fundamental processes that may clarify this correlation, as well as the different variables that could influence the likelihood, such as the length and strength of night shift employment, a person's susceptibility, and the possible impact of personal lifestyle decisions. Furthermore, this evaluation will emphasize the significance of these results on both public health strategies and job procedures. It is crucial to comprehend the correlation between working night shifts and the risk of T2DM in order to address potential health impacts and enhance the overall welfare of individuals working non-traditional hours. Thus, the article aims to offer a thorough evaluation of the current understanding of the subject matter, compiling important discoveries, pinpointing deficiencies in existing literature, and proposing ideas for future areas of study. Our goal is to add to the existing research on night shift work and T2DM, which will help us gain a better understanding of the health consequences of current work schedules. This will also aid in the creation of tactics to reduce potential risks.

## 2 Glucose metabolism and diabetes mellitus

Diabetes mellitus is a group of metabolic conditions characterized by chronically elevated levels of blood sugar due to various underlying disturbances in the regulation of glucose (27). In a healthy individual, the body regulates glucose through hepatic glucose production (gluconeogenesis) and its utilization in both insulin-dependent tissues (e.g., muscle, fat) and insulin-independent tissues like the brain, which relies on insulin-independent glucose transporters (28–30). Pancreatic β cells constantly and, when necessary, promptly react to the presence of glucose, generating, and releasing insulin. The hormone insulin is vital in aiding the absorption of glucose by tissues throughout the body (31, 32). Moreover, it hinders the liver's production of glucose and the metabolism of fats in fatty tissue (33). The impact of insulin's effects on target tissues, referred to as insulin sensitivity, is affected by multiple physiological elements, where obesity is considered a primary determining factor. Insufficient tissue reaction, referred to as insulin resistance, results in reduced absorption of glucose (34–36).

T1DM is mainly caused by the loss of pancreatic β-cells, resulting in a deficiency of insulin. On the other hand, T2DM, which is the most prevalent type, is distinguished by an inability to respond to insulin and a partial lack in the production of insulin, typically resulting in an insufficient rise in insulin levels (37, 38). Nonetheless, there are certain people who may display a more prominent scarcity in their production of insulin. The emergence of T2DM comprises a multifaceted interaction between hereditary and external influences, wherein abdominal obesity plays a crucial role as a risk factor (39). The various stages of T2DM can be clearly identified in clinical terminology. Impaired glucose regulation refers to a state between having normal glucose tolerance and the full development of T2DM. This ailment can be identified by elevated fasting glucose or impaired glucose tolerance, usually evaluated using an oral glucose tolerance test (40, 41). Crucially, this particular condition of the body's metabolism serves as a reliable warning sign for the eventual development of T2DM (42). The metabolic syndrome, also known as insulin resistance syndrome, comprises of two important aspects: insulin resistance and glucose intolerance. The term “syndrome” refers to a compilation of various risk factors associated with maintaining optimal cardiometabolic wellbeing. Although various standards have been suggested to delineate the illness, professionals generally agree that it includes insulin resistance, glucose intolerance, excess weight around the waist, high blood pressure, and abnormal levels of lipids (including high levels of triglycerides and low levels of high-density lipoprotein cholesterol) (43–45). Other linked characteristics include high levels of uric acid, a state of swelling and blood clotting, excessive leptin levels, and the presence of small amounts of protein in the urine (46). T2DM is a considerable burden in terms of illness and is considered one of the primary contributors to mortality in multiple countries (47). The major cause of the elevated death rate is largely connected to the consequential and serious health issues it triggers, specifically cardiovascular disease. In addition, with the ongoing rise of obesity at a global level, it is predicted that the incidence of T2DM will also go up (48).

By 2017, the number of deaths caused by T2DM in individuals over the age of 20 had risen to 5 million, a significant increase compared to the 665,000 deaths recorded in 1990 (49, 50). There are around 451 million individuals, 18 years old and above, who have been diagnosed with T2DM across the globe according to current calculations (49). Along with the growing occurrence, T2DM and its related issues place a significant financial burden on healthcare systems worldwide (51). According to recent research, it is estimated that Canada will experience a significant rise in the number of T2DM cases over the course of 10 years, with a projected count of 2.16 million new diagnoses. The increase is paired with healthcare expenses totaling $15.36 billion, primarily due to costs associated with hospital stays and purchasing prescribed drugs (52).

## 3 The epidemiological studies on the association between night shift work and the risk of T2DM

Night shift work is a prevalent aspect of contemporary societies, and a growing body of epidemiological research underscores its negative health impacts, particularly its link to the development of T2DM. Table 1 (refer to the table for detailed study characteristics) presents essential epidemiological research investigating this connection. Multiple studies demonstrate a relationship between night shift work and an increased incidence of T2DM. A longitudinal study in Japan followed 2,860 male workers for 8 years, classifying T2DM based on glycated hemoglobin levels or physician diagnosis. While initial relative risks for two-shift (RR = 1.73) and three-shift (RR = 1.33) workers compared to fixed day shifts were not statistically significant after adjustment, a notable and statistically significant increase in T2DM likelihood (RR = 2.01) was observed among two-shift workers when compared to white-collar employees, suggesting a varying risk based on specific work schedules (53). Further reinforcing this link, a 15-year cohort study of 19,873 Danish nurses revealed that those working night or evening shifts had a significantly higher risk of developing T2DM compared to day shift workers, a finding that remained robust even after accounting for BMI. The greatest risk was specifically associated with ongoing night shift work Hansen et al. (54). Similarly, a longitudinal study in Stockholm, Sweden, involving ~28,000 nurses and nursing assistants, found that individuals with night shifts in the past year or frequent/rigorous shift work (over 120 afternoon and/or night shifts in the prior year) had a significantly higher chance of developing T2DM. This study also noted an increased risk from extended periods of three or more consecutive overnight shifts and multiple years of permanent night shifts Viklund et al. (15).

**Table 1:** An overview of epidemiological studies investigating the potential link between night shift work and the risk of developing type 2 diabetes mellitus.

**Country**	**Design**	**Follow-up years**	**Population**	**Gender**	**Age**	**Shift work definition**	**Shift work type**	**Key findings related to T2DM**	**Ref**
Japan	Cohort	8	2,860 men from a sash and zipper manufacturing plant	Male	Not specified (working age)	Three distinct cohorts: consistent daytime blue-collar, rotating shifts blue-collar, white-collar. Rotating shifts involve two or three shifts, which may include night shifts, with a weekly clockwise rotation.	Two-shift, three-shift, fixed daytime, white-collar	Two-shift workers had a 1.73 relative risk of T2DM (compared to fixed day shifts), and three-shift workers had 1.33. Two-shift workers showed a notable rise in T2DM likelihood (RR = 2.01) when compared to white-collar employees, but no such trend for three-shift or fixed daytime blue-collar workers.	(53)
Brazil	Cross-sectional (Initial data from a longitudinal study)	NA (baseline analysis)	15,105 public sector employees (ELSA-Brasil cohort)	Both	35–74	Defined as prior involvement in night shifts for a subset of the population.	Night shift	Increased BMI and waist circumference linked to night work in men. After adjustments, night shifts associated with increased fasting plasma glucose, glycated hemoglobin, and 2-h plasma glucose in women.	(56)
Denmark	Cohort	15	28,731 women nurses (Danish Nurse Cohort)	Female	51.4 ± 5.4 (at baseline)	Self-reported work schedules: day, evening, night, or rotating shifts.	Evening, night, Rotating	Nurses working night or evening shifts had a higher risk of developing T2DM compared to day shifts, even after adjusting for BMI. Greatest risk linked to ongoing night shift work.	(54)
USA	Cohort	22–24	143,410 female nurses (Nurses' Health Study I & II)	Female	30–55 (at baseline)	Rotating night shift: working at least three-night shifts per month, in addition to day and evening shifts within the same monthly period.	Rotating night shift	T2DM risk was higher among female nurses with rotating night shift work and unhealthy lifestyles. Combined risks were greater than the sum of individual factors.	(55)
Brazil	Cohort	3.8 (average)	8,636 participants from ELSA-Brasil (subset of 15,105)	Both	Not specified (within 35–74 range)	Long-term exposure to night shifts, specifically defined by participants selecting “day shifts only, night shifts only, or mixed.”	Night shift	Women working night shifts for ≥10 years had a higher likelihood of developing T2DM (crude incidence rate 2.26 per 100 person-years), even after adjusting for age, education, work hours, and BMI. This connection slightly diminished when exercise was considered. Not statistically significant in men.	(57)
Taiwan	Retrospective Cohort	17	Healthcare workers at a tertiary medical facility	Both	20–65	Day (8:00–16:00), evening (16:00–24:00), and night (0:00–8:00) shifts. Night shift workers categorized by hours: < 17, 17–45, ≥46 hours/month.	Day, evening, night	Overall, T2DM prevalence in night shift employees was not significantly greater than day workers. However, a direct correlation was found between extended night shift hours (≥17 hours/month) and an elevated likelihood of developing T2DM, with risk increasing proportionately.	(58)
Sweden	Longitudinal Cohort	4 (monitoring period 2013–2017)	~28,000 nurses and nursing assistants (Stockholm)	Both	Not specified	Defined by detailed personal information regarding daily schedule and hours worked. Categories included past year night shifts, frequent/rigorous shift work (≥120 afternoon/night shifts in prior year), day and afternoon shifts, consecutive night shifts, and permanent night shifts over multiple years.	Night, rotating, day/afternoon, permanent night	Individuals with past year night shifts and frequent/rigorous shift work had a significantly higher chance of developing T2DM. Higher chance from extended periods of ≥3 consecutive overnight shifts and multiple years of only working at night.	(15)

The interplay between shift work and lifestyle factors also plays a crucial role. Research by Shan et al. (55) on female nurses (Nurses' Health Study I & II) demonstrated that rotating night shift work, when combined with unhealthy lifestyle behaviors (e.g., smoking, low physical activity, poor diet, high BMI), led to a compounded T2DM risk that was greater than the sum of the individual risks. This highlights a significant opportunity for T2DM prevention through health-conscious lifestyle changes, especially for individuals in fluctuating night shifts. Several studies suggest a sex-specific susceptibility to the metabolic consequences of night shift work. The Brazilian Longitudinal Study of Adult Health (ELSA-Brasil) initially showed increased BMI and waist circumference linked to night work in male participants (56). However, a separate and more focused longitudinal analysis from the same ELSA-Brasil cohort specifically examined the effects of long-term night shift exposure on T2DM. This study found a higher likelihood of developing T2DM in women who had worked night shifts for at least a decade, even after adjusting for factors like age, education, work hours, and BMI. This association was slightly diminished when exercise was considered but notably, the findings were not statistically significant in the male population. These results suggest that the impact of chronic night shifts on T2DM risk might be more pronounced in females than males Silva-Costa et al. (57). While the bulk of the evidence points toward an increased T2DM risk with night shift work, some studies present more nuanced findings or apparent contradictions. For instance, a retrospective cohort study of 7,081 healthcare workers in Taiwan found that, overall, the prevalence of T2DM in night shift employees was not significantly greater than in day workers. However, this study also identified a direct correlation between extended hours on a night shift (specifically ≥17 h or ≥46 h per month) and an elevated, proportionately increasing likelihood of developing T2DM Chen and Yang (58). This seemingly contradictory finding might be influenced by the “healthy worker effect,” where individuals who continue night shift work may represent a healthier subset of the population (59). This could potentially weaken the observed link between longer night shift hours and T2DM, leading to an underestimation of the true risk for those working extended night shifts Chen and Yang (58). Collectively, the epidemiological research strongly suggests that shift work, particularly night shift work, is a potential contributor to a higher likelihood of developing T2DM. While individual study findings present subtle differences and complexities, particularly regarding sex-specific effects and the impact of the “healthy worker effect,” the overall evidence emphasizes the importance of addressing the potential dangers of shift work and implementing preventative measures, such as lifestyle interventions, for individuals in these occupations. These studies also highlight the need to consider the intricacies and variations in risk across different populations and work environments.

## 4 Underlaying mechanisms involved in increased risk of T2DM among night shift workers

### 4.1 Circadian rhythms and T2DM

Circadian rhythms refer to the recurring 24-h patterns that occur in all forms of life, regulating a diverse range of biological activities (60). The main internal clock in mammals is mostly found in the suprachiasmatic nucleus (SCN) located in the front part of the hypothalamus. Moreover, there are peripheral clocks present in multiple areas of the brain, as well as nearly all peripheral tissues and organs (61). The biological timekeeper, consisting of fundamental timekeeping genes such as clock, bmal, and per (1, 2, and 3), cry (1 and 2), rev-erba, and ROR, functions by maintaining a constant cycle of transcription and translation through a mechanism of self-regulated feedback that spans ~24 h. Any disruption in these fundamental genes responsible for regulating the body's internal clock can increase a person's vulnerability to developing T2DM, gaining excessive weight, and experiencing metabolic syndrome (62, 63). The hormone cortisol exemplifies the importance of circadian rhythmicity in metabolic regulation. Cortisol normally follows a distinct pattern, peaking upon waking and reaching its lowest point at sleep onset. This rhythm plays a crucial role in glucose homeostasis throughout the day. When this pattern is disrupted, as in night shift work, metabolic dysregulation often follows. Loss of synchrony between circadian clocks and hormonal rhythms directly contributes to metabolic disorders. Evidence from shift workers demonstrates that this desynchronization is associated with obesity, depression, and T2DM (64). The implications extend beyond simply feeling tired—circadian misalignment fundamentally alters how the body processes nutrients, particularly glucose. The SCN transmits synchronized time-of-day data to the entire brain through direct or indirect neural pathways, such as the parasympathetic and sympathetic nervous systems. It can also influence the release of factors such as insulin and melatonin (65). It is significant to mention that sympathetic nerve activity triggers the production of glucose, whereas parasympathetic nerve activity promotes the formation of hepatic glycogen in the liver. Individuals with prediabetes display heightened sympathetic activity before encountering insulin resistance, which differentiates them from their healthy counterparts (66).

Humans typically differ from most other mammals in terms of their sleep habits. Unlike other animals, humans tend to have uninterrupted sleep for a stretch of 7–9 h, leading to a longer period of fasting overnight (67). The quality of sleep significantly affects the functioning of pancreatic β-cells and the body's ability to process insulin (68). Interestingly, even though there is a prolonged period of not eating during overnight sleep, blood sugar levels stay consistent or experience only slight drops. When people are both alert and abstaining from eating while lying down without moving, their glucose levels typically decrease by an average of 10 to 20 mg/dL within a 12-h period (69–71). As previously explained, glucose serves as the main source of energy and is broken down by practically all recognized living beings to sustain their survival (72). Due to the fact that an organism's energy requirements change according to the time of day, the fluctuations in glucose metabolism during the day can be attributed, at least in part, to the changing utilization of glucose on a daily basis (73). The levels of glucose in the blood are influenced by the coordinated regulation of how much glucose is consumed through food and how much is produced by the liver, as well as how much is used by the muscles and adipose tissues. Many researchers have documented a consistent cycle in the levels of glucose found in the bloodstream over the course of a day. The first suggestion of the involvement of the SCN in regulating glucose levels was discovered through studies showing that damaging the bilateral SCN significantly disrupted the regular patterns of both plasma glucose and insulin. These lesions also eliminated the distinct day-to-night response to 2-deoxy-glucose, an inhibitor of glucose utilization, as noted in the study by Nagai et al. (74).

The baseline glucose levels in mild fasting conditions, such as before meals, show a diurnal pattern in healthy animals and human participants, influenced by regular light and dark cycles. This rhythm reaches its peak upon waking and hits a trough during sleep (75). It is significant to note that this daily cycle is not exclusively caused by a person's eating habits, as it remains present even when fasting takes place at varying times (76). The use of isotope tracers has revealed a concordant daily pattern in the production of natural glucose within the bodies of individuals who are in good health (77). This discovery suggests that the daily variation in glucose levels is mainly connected to the endogenous glucose production. The liver is essential to the process of EGP, as it either breaks down glycogen or creates glucose through the process of gluconeogenesis. Corresponding to this, the substance of liver glycogen demonstrates a daily pattern that contradicts the daily baseline levels of glucose observed in humans (78). The natural daily rhythm of normal glucose levels is disrupted in animals that have damage to the SCN. The removal of SCN results in a heightened level of c-Fos expression in ARC neurons caused by 2-deoxy-glucose, which further exacerbates hyperglycemia after waking up. The implication is that the SCN-ARC projection could potentially have a significant impact on maintaining the daily glucose cycle by aiding in the counteractive response to hypoglycemia (74, 79). Unlike people who are in good health, individuals who have prediabetes experience an alteration in the timing of their fasting blood sugar fluctuations, as their highest blood sugar level occurs later in the day. After analyzing hypothalamic tissue taken from deceased individuals with T2DM, it was found that there was a decrease in the amount of neurons in the SCN region compared to those without the condition. The act of observing implies that there is a chance that people with T2DM may experience disruptions in their SCN (80).

The daily pattern of changes in glucose levels after eating is significantly different from the initial levels of glucose before eating. In individuals with good health, consuming the same meal leads to a larger rise in glucose levels at dinner than at breakfast (81). According to research conducted on humans using the oral glucose minimal model or hyperinsulinemia-euglycemic clamp, it has been observed that insulin sensitivity is at its peak in the morning and gradually declines throughout the day, reaching its lowest level during sleep. The significant changes in EGP inhibition via insulin demonstrate notable fluctuations throughout the day (82). The SCN plays a vital role in the fluctuations of glycemic responses to meals based on the time of day. When the SCN is damaged, the diurnal pattern in glucose tolerance is abolished. Moreover, it is essential for the correct functioning of clock genes in the SCN in order to maintain the daily fluctuations in glucose tolerance and hepatic insulin sensitivity (83, 84).

**Figure 1:**
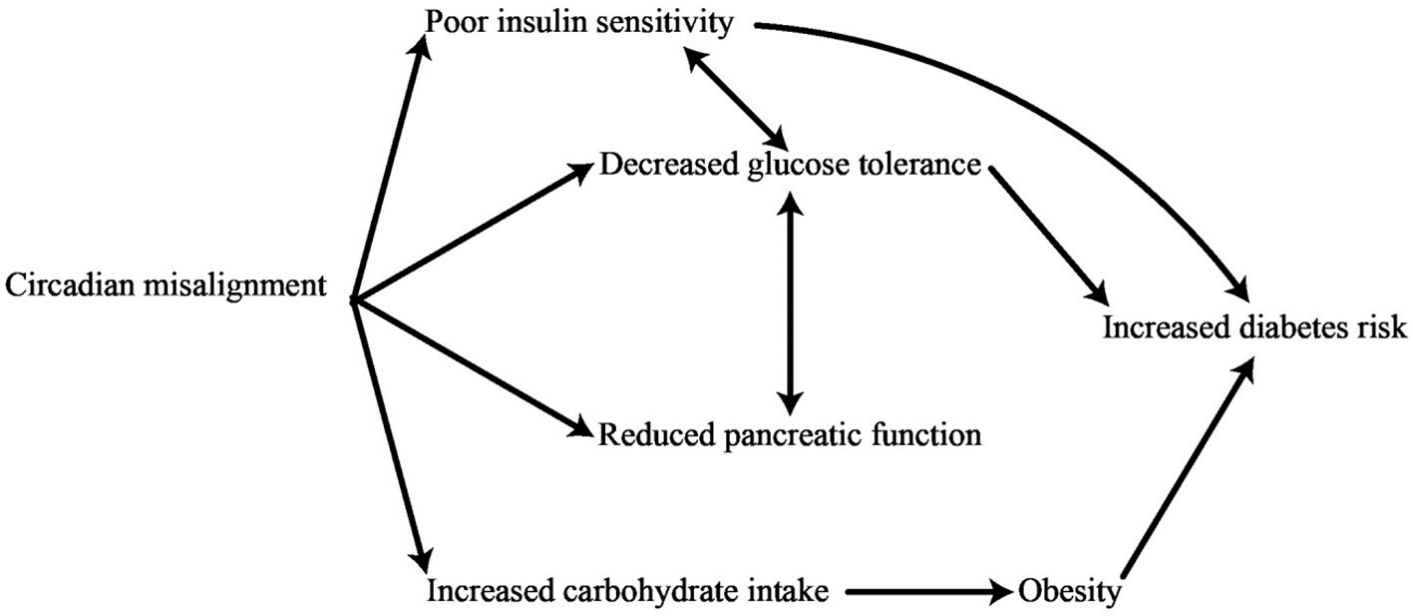
Physiological impacts of disruptions in circadian rhythms on the risk of developing diabetes.

It may seem contradictory to have both elevated baseline glucose levels and increased insulin sensitivity upon awakening, but this can be explained from an evolutionary perspective. When the body enters a state of sleep, the need for EGP to ensure the blood sugar levels remain steady increases. The need for energy is highest right after waking up and right before a meal, since the body requires glucose to function effectively for tasks such as thinking and moving while searching for food on an empty stomach (85, 86). Insulin entering the body after a meal unlocks this capability and finalizes the change from elevated pre-meal glucose production to reduced post-meal glucose production. Therefore, the SCN clock is crucially essential in regulating the predictive management of liver response to insulin, working alongside insulin signaling to coordinate the daily cycle of glucose processing in typical physiological circumstances (87). When a person is in a normal physical condition, both the initial amount of endogenous glucose production and its responsiveness to insulin-induced decrease reach their highest levels upon awakening. It should be emphasized that while the autonomous nervous system (ANS) plays a key role in regulating glucose rhythms as discussed previously, the SCN also has the ability to impact the daily liver glucose rhythm through the release of certain factors (88). As such, the regulation of blood sugar levels is influenced by both the SCN as well as the peripheral clocks found in different tissues. Recent studies indicate that disturbances in the body's natural patterns of biological processes can have an effect on the way metabolic pathways function. The internal system for keeping time is intricately connected to the circadian control of glucose metabolism and the functioning of insulin. In this light, changes in the daily pattern of glucose tolerance serve as an initial sign of T2DM. In contrast to healthy individuals, those who have prediabetes show improved management of glycemic levels when consuming a bolus dose of glucose or a meal in the evening rather than in the morning (89). The study of disease patterns shows that the disruption of the body's internal clock can be caused by various factors, such as inconsistent eating habits, minimal exposure to daylight, higher exposure to light at night due to shift work, lack of sleep, having a late sleep schedule, social jet lag, and other elements. These factors are linked to an increased risk of developing T2DM (90). The internal biological clocks within pancreatic β-cells are responsible for controlling the timing of gene transcription related to important processes such as glucose metabolism, protection against oxidative damage, and insulin release. Figure 1 strengthens the correlation between circadian rhythms and T2DM (63).

The regulation of carbohydrate, lipid, and protein metabolism through circadian rhythms is dependent on a collective control by oscillators (91). The circadian rhythm plays a crucial role in controlling the expression and activities of crucial metabolic components, such as receptors, transporters, and enzymes. Disruptions in peripheral clocks located in a range of organs (such as the liver, muscles, pancreas, adipose tissue, intestines, kidneys, etc.) contribute to altering metabolic traits (92). The levels of glucose tolerance in humans vary on a daily basis. The occurrence of this phenomenon is a direct consequence of the everyday fluctuations in peripheral insulin sensitivity, which are caused by essential internal cellular mechanisms that regulate the intake of glucose. The liver releases glucose while the body is at rest, but during periods of physical activity, other organs absorb glucose (93). Individuals who are overweight or advanced in age may experience patterns that are diminished, postponed, or nonexistent. The dawn phenomenon, which describes the rise in blood sugar levels in the morning, can be attributed to changes in the body's response to insulin (94). Disrupting the function of clock genes through changes in their genetic makeup leads to a decrease in the production of TBC1D1, GLUT4, and hexokinase, resulting in a deficiency in the ability to use glucose when stimulated by insulin. Metabolic detectors, including Sirt1, AMPK, PGC-1a, NAD, and FAD, have the function of transmitting the cell's energy level to the primary circadian rhythm (95). The absence of a circadian rhythm in β-cells has been found to decrease the conversion of substrates, the production of ATP, and the transportation of vesicles, ultimately resulting in diminished insulin secretion (96). The balance between NAD and NADH is vital in dictating the interaction of the NPAS2-CLOCK partnership with specific genes in the body. The circadian rhythm of metabolism is controlled by transcription factors, such as DBP, Rev-Reba, PPARg, HLF, and TEF, which play a crucial role in regulating different metabolic pathways (97). If the circadian rhythms are disturbed, it can cause an improper regulation of mitochondrial function, resulting in a higher production of reactive oxygen species (ROS) (98). ROR proteins and PGC-1a play a crucial role in controlling the diurnal rhythms of lipid and glucose metabolism (99). Both the MTP found in the intestines and the MTP found in the liver show cyclical patterns of activity, peaking at the same time as the levels of lipids in the blood. In addition, the activity of clock genes in the intestines controls the cyclical production of MTP, apolipoprotein A IV, and nocturnin, which are all essential components in the process of absorbing lipids (100). REV-REBa's circadian function plays a pivotal role in regulating the periodic production of enzymes responsible for synthesizing lipids (99).

The naturally occurring patterns of physiological processes, such as the production of certain enzymes involved in the breakdown of fats, namely HMG CoA reductase, hormone-sensitive lipase, carnitine palmitoyltransferase 1, and medium-chain acyl-CoA dehydrogenase, are known as circadian rhythms. Genetic mutations in the clock gene of mice lead to alterations in the periodic manifestation of genes that control the production of triglycerides (TGs) and the breakdown of fats. The Clock gene plays a significant role in reducing the activity of microsomal triglyceride transfer protein (MTP) by specifically attaching to the Hepatocyte Nuclear Factor4 located at the MTP promoter site. This process is crucial for maintaining daily variations in plasma triglyceride levels, achieved through the regulation of adipogenesis by BMAL1 and the control of important factors involved in both adipogenesis and lipogenesis (101). The interference with genes regulated by the internal clock (CCGs) that are located after the main clock mechanism has an impact on the processing of lipids. Mice that were put on a low-protein diet encountered disruptions in the regular fluctuations of plasma glucose, triglycerides, and insulin levels. Restricting protein intake can cause alterations in the peripheral clock, highlighting the importance of appropriate nutrition in regulating the biological clock. Moreover, inadequate protein consumption throughout pregnancy has been discovered to impede the body's daily rhythm system and affect several aspects associated with metabolic syndrome. Moreover, peripheral oscillators have experienced interruptions as a result of neuronal alterations within the SCN (102).

### 4.2 Melatonin and diabetes mellitus

As mentioned earlier, the circadian system has developed to produce ~24-h patterns in both behavior and bodily processes. This ability allows the organism to anticipate and accommodate the recurring changes in its surroundings, such as the cycle of light and darkness resulting from the Earth's rotation. The internal biological rhythms found in mammals are a product of a complex system of oscillators. This includes the primary circadian pacemaker located in the hypothalamic SCN and smaller clocks found in various organs, tissues, and cells throughout the body. The primary means of synchronization for the SCN is through light input, which is achieved through direct photic signals that originate in the retina and are then transmitted to the SCN via the retinohypothalamic tract. The primary function of the SCN is to align and coordinate the peripheral clocks by utilizing signals from neurons, behaviors, body temperature, and hormones. The SCN is responsible for producing melatonin, which is recognized as one of the most commonly recognized hormonal signals (103). The pineal gland in the brain of mammals is responsible for the production and release of melatonin at night, following a regular cycle known as the circadian rhythm. It acts as a middleman for regulating internal biological cycles and has an influence on various bodily processes. Melatonin displays a multitude of beneficial properties including anti-oxidative, anti-inflammatory, and anti-aging effects. Additionally, it plays a crucial role in treating a variety of sleep disorders (104, 105). The main function of Melatonin on a physiological level is to convey important details regarding the length and timing of the night to the entire body (73). The synthesis of melatonin in mammals is governed by an intricate sympathetic pathway that stems from the SCN to the pineal gland. The primary determinants of the amount of melatonin found in the bloodstream are the current phase of the circadian rhythm and the amount of light exposure. For people who follow a schedule during the day, there is a rise in their melatonin levels a couple of hours before their usual bedtime. These levels stay high throughout the night, but then decrease in the early hours after they typically wake up. For the rest of the day, the levels remain very low. When exposed to light at night, there is a swift and proportional decrease in melatonin levels, whereas being in darkness during the day does not trigger the production of melatonin. The regulation of both the circadian rhythm and the suppression of melatonin by light are controlled by the SCN, which receives visual signals from specialized retinal ganglion cells that are sensitive to light through the retinohypothalamic tract (106, 107).

Famous for its function as a coordinating factor or timekeeper, melatonin is actively engaged in coordinating the cyclical patterns in different physiological functions within the body. The glucose tolerance of individuals in good health follows a consistent daily trend, with a gradual decrease in tolerance as the day goes on (67). The regular pattern continues to exist even when the impact of external and personal factors is eliminated, indicating that it is regulated by one's internal body clock (108). Melatonin, known for its elevated levels during the night and its ability to lower glucose tolerance, may be a contributing factor to the decline in glucose tolerance during nighttime in human beings (109). Furthermore, numerous well-established processes governed by regulatory pathways, such as the autonomic nervous system, changing hormone levels (such as glucocorticoids), and self-regulating peripheral clocks with the ability to affect organ function (such as insulin secretion from pancreatic islets and insulin sensitivity of adipocytes), play a role in this phenomenon (110, 111).

Over the past few years, there has been a significant amount of research done on the effects of melatonin on insulin release and the regulation of glucose levels in the body. Melatonin carries out its actions through two different forms of receptor proteins located on the membrane, which are called MT1 (also known as Mel1a and encoded by MTNR1A) and MT2 (also known as Mel1b and encoded by MTNR1B). These specific receptors fall into the category of G-protein coupled receptors and have the ability to convey the effects and effects of melatonin through their interaction with Gi proteins that are sensitive to pertussis toxin. This communication leads to the blocking of adenylate cyclase (AC), which eventually causes a decline in cellular cAMP concentrations. As a result, these occurrences have an impact on the subsequent objectives (112). Moreover, it is crucial to acknowledge the presence of a different binding site that has a strong attraction to melatonin, specifically the MT3 subtype (Mel1C). This particular variation has been effectively reproduced from non-mammalian creatures such as zebrafish, Xenopus, and chicken. Despite extensive research, this subtype has yet to be detected in mammals (113). In the world of mammalian species, the distinct equivalent of MT3 can be found in the form of GPR50, a receptor that has yet to be fully understood or classified. It should be noted that despite being mentioned, GPR50 does not possess the ability to bind to melatonin, and its role in human biology is still unknown (110). Research revealed that when melatonin was administered through an intracerebroventricular injection, it triggered the activation of hypothalamic Akt and inhibited hepatic gluconeogenesis. The discovery suggests that melatonin is involved in promoting communication between the hypothalamus and the liver. This subsequently implied a possible connection between the decreased amounts of melatonin found in those with T2DM and disruptions in the body's circadian metabolic functions (114). On the other hand, in contrast to the widely accepted idea that melatonin plays a key role in regulating glucose metabolism, there is a decrease in melatonin production when there is high blood sugar. This disability is linked to a potential initial decline in the level of β-adrenergic receptors, which play a vital role in kickstarting the process of producing melatonin. It is widely believed that this decrease will cause even more disruption to the control of glucose levels and intensify the progression of T2DM (115).

Since insulin plays a critical role in regulating metabolism, it is imperative to focus on how melatonin affects the secretion of insulin. Melatonin decreases the secretion of insulin (116). Numerous studies have affirmed that melatonin's role in regulating insulin release is achieved by engaging in communication with both the MT1 and MT2 receptors. By utilizing the melatonin receptors and the accompanying signaling cascade facilitated by the Gi-proteins, melatonin has the ability to suppress the AC/cAMP system (in the instances of MT1 and MT2) and the GC/cGMP system (with a focus on MT2), effectively decreasing the levels of insulin released. On the other hand, if melatonin receptors bind with Gq proteins, this triggers phospholipase C to initiate the production of insulin through the IP3-signaling pathway. However, it should be emphasized that melatonin predominantly has a detrimental effect on the release of insulin (117).

**Figure 2:**
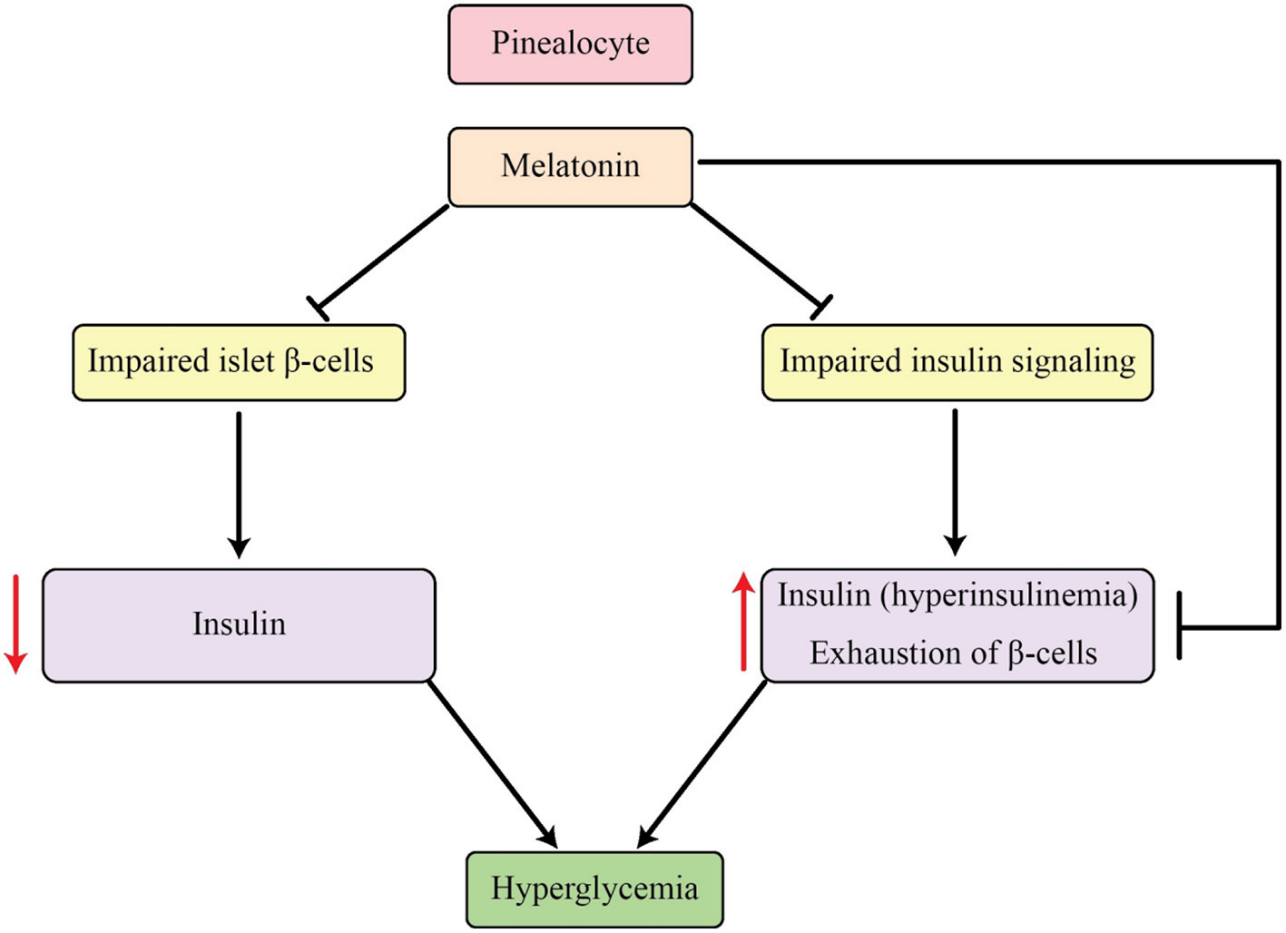
Influences of melatonin on the regulation of insulin and glucose levels in the body.

Melatonin's well-established ability to hinder insulin is marked by a contradictory rhythmic fluctuation between these two hormones, a phenomenon that is also apparent in cases of T2DM (118). In the initial phases of T2DM, there is an increase in insulin secretion but a decrease in melatonin levels. Conversely, in type 1 diabetes, there is a reverse connection between these two factors (119, 120). The link between T2DM and this symbiotic connection is primarily attributed to catecholamines, specifically NE, according to current theories. Catecholamines can stimulate the production and release of melatonin through receptors like adrenoceptor β1-cAMP or α1-IP3 cascade, while at the same time blocking insulin secretion through α2 receptors. Additionally, α2 receptors have the ability to decrease the release of norepinephrine by acting on nerve terminal autoreceptors. These mechanisms may provide an explanation for the diminished plasma catecholamines, reduced melatonin, and elevated insulin levels observed in T2DM, as elucidated in studies by Frese et al. (121) and Peschke et al. (122). It is believed that melatonin serves as a protective measure against the strain on β-cells caused by T2DM by inhibiting insulin secretion, as the depletion of these cells is linked to the development of the disease. As a result, this process provides a safeguard against the progress of T2DM. Therefore, the decrease in melatonin levels and the rise in incidence of insulin resistance and T2DM among aging individuals can be acknowledged as a result of melatonin's defensive effects on β-cells and their performance (123). Within the framework of type 1 diabetes, the increased quantities of melatonin serve as a protective measure utilized by the body to counter the strain caused by diabetes, thereby reducing the harm caused to β-cells due to oxidative stress (124). As a whole, melatonin has the ability to improve the functioning of β-cells and hinder the development of T2DM (refer to Figure 2 for a visual depiction of this).

### 4.3 Sleep deprivation and diabetes mellitus

**Figure 3:**
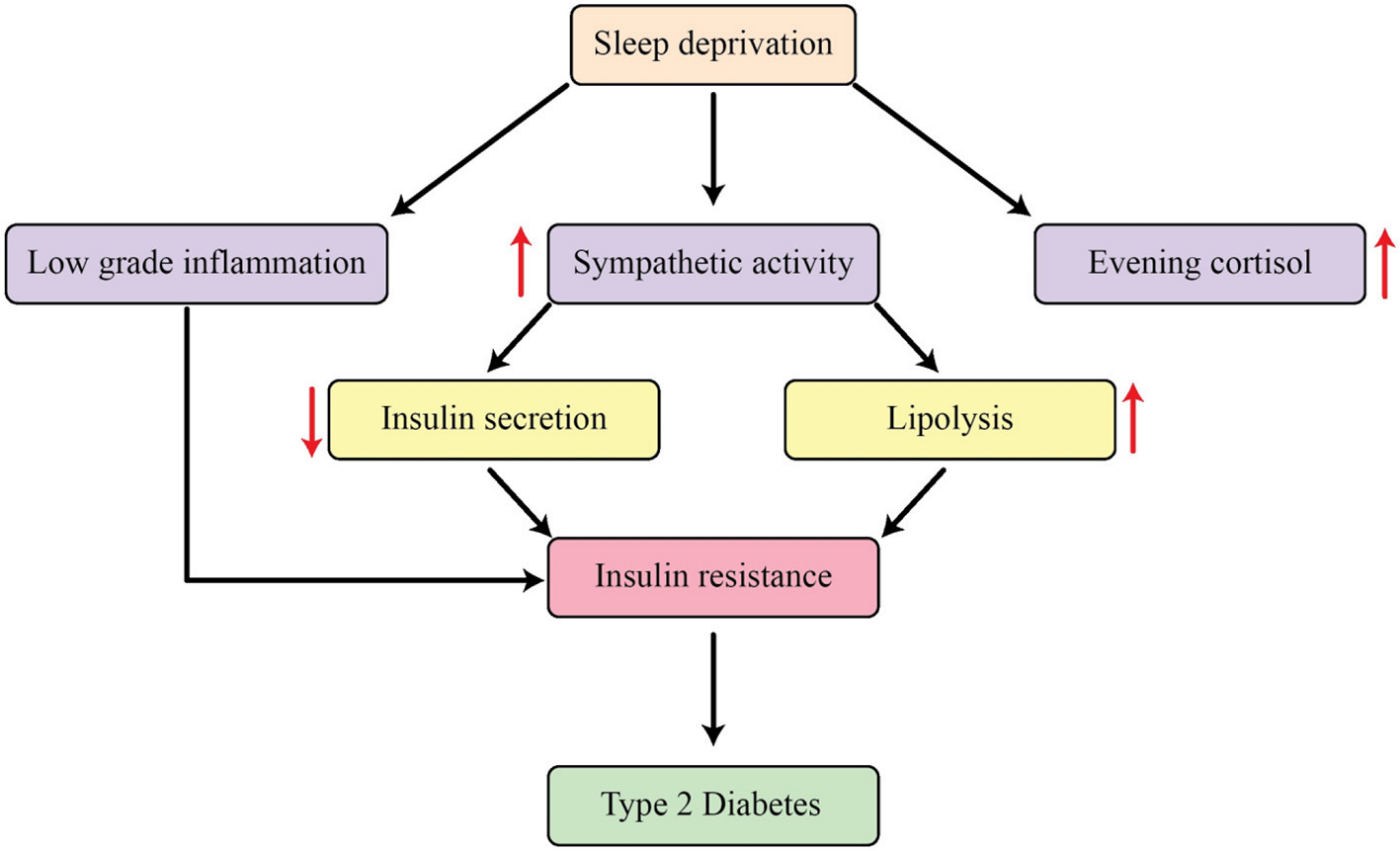
The mechanisms that explain how sleep restriction affects metabolic processes in the body.

The results of studies performed in controlled laboratory environments with individuals who are in good health have confirmed a correlation between decreasing the amount of sleep and a decrease in the body's ability to respond to insulin (125). Research has shown a marked decline in the brain's consumption of glucose when deprived of sleep for extended periods (126). Additionally, restricting the amount of sleep resulted in a decrease in the ability of subcutaneous fat cells to respond to insulin in healthy individuals (127). Multiple intermediate processes could be involved in this correlation (Figure 3). Sleep restriction is associated with an increase in activity of the sympathetic nervous system. The sympathetic nervous system has a profound impact on a variety of bodily processes, including the secretion of insulin and glucagon, the development of muscle insulin resistance, and the function of adipocytes (128). The initiation of the hypothalamic-pituitary-adrenal (HPA) axis may also contribute to this occurrence. Research has shown a strong correlation between lack of sleep and increased levels of salivary and serum cortisol, as well as the absence of the usual decrease in serum cortisol during the evening (129). Several research projects examining the effects of limited sleep have shown that cortisol levels are significantly higher during the later parts of the day, and these rises may play a role in the development of insulin resistance. However, it is important to acknowledge that while the reversal of the normal cortisol rhythm has been proven to cause insulin resistance, there was not always a direct correlation between changes in cortisol levels and changes in insulin sensitivity after periods of sleep deprivation (130). Extended GH release during sleep deprivation may add to insulin resistance in the morning (131). Furthermore, sleep restriction consistently leads to an increase in inflammatory markers, with notable variations in the levels of IL-1β, IL-6, IL-17, TNF-α, hsCRP, as well as a rise in leukocyte and monocyte counts. Insulin resistance has been linked with these markers of inflammation (132).

Furthermore, an increase in sympathetic activity causes an increase in the breakdown of fats, leading to an increase in levels of free fatty acids in the bloodstream. This can ultimately lead to the buildup of fat in the liver and muscles, leading to the development of insulin resistance. The concept of dysfunctional fat cells has gained recognition as a crucial connection between inadequate sleep and negative metabolic effects. The HPA axis activation and heightened sympathetic activity are both believed to contribute to the development of a condition known as “adipocyte insulin resistance” (133). Decreased insulin signaling in adipocytes could potentially trigger the breakdown of fats, leading to the release of NEFA, which could then worsen insulin resistance. Elaborate and continuous growth hormone production during the night and elevated early-morning levels of noradrenaline may play a role in this mechanism, further supported by their close correlation with the increase in nighttime NEFA levels. As previously mentioned, insufficient sleep can have a significant impact on the hormones responsible for regulating appetite, food consumption, and energy usage. This can ultimately lead to indirect effects on glucose metabolism and increase the likelihood of developing T2DM by promoting weight gain and ultimately causing the body to become resistant to insulin (131).

### 4.4 Night shift work and metabolic disruption

Experimental studies have isolated the specific effects of circadian misalignment on glucose metabolism. Research has demonstrated that both the endogenous circadian system and circadian misalignment independently affect glucose tolerance in shift workers. Postprandial glucose responses were 6.5% higher at 8:00 p.m. than at 8:00 a.m., representing a circadian phase effect independent of behavioral factors. Furthermore, circadian misalignment itself increased postprandial glucose by an additional 5.6%, independent of both behavioral and circadian phase effects (134). These glucose variations appear to result from different insulin mechanisms. In controlled studies, circadian misalignment increased markers of insulin resistance and inflammation independently of sleep loss. Particularly striking was the observation that in male participants exposed to circadian misalignment, both the reduction in insulin sensitivity and the increase in inflammation doubled compared to those maintaining regular sleep schedules (135). This demonstrates that misalignment itself, beyond sleep deprivation, directly impacts metabolic health.

Night shift work triggers multiple pathophysiological mechanisms that collectively increase T2DM risk. Melatonin, the primary marker of the circadian system, has multiple biological actions relevant to cardiometabolic health, including modulation of oxidative stress, inflammation, and vascular function through its receptors (136). Disruption of melatonin signaling during night shifts likely contributes to metabolic dysregulation. Gene expression changes represent another important mechanism. NSW has been shown to modify gene expression and functional readouts in different tissues and organs, which appear to translate into persistent cardiometabolic and endocrine alterations (137). These molecular changes help explain how chronic exposure to night shift work can lead to lasting metabolic consequences even during periods away from work. Night shift work often triggers behavioral changes that compound circadian disruption. Mistimed feeding—eating during the biological night when the digestive system is not optimally prepared to process nutrients—frequently accompanies night shift work (137). This combination of circadian misalignment and inappropriate meal timing synergistically increases metabolic risk.

Sleep disturbances represent another critical pathway. Night shift workers commonly experience reduced sleep quality and quantity, which independently contribute to insulin resistance and glucose dysregulation. Total sleep time and sleep efficiency are frequently compromised in shift workers, creating a vicious cycle of circadian disruption, poor sleep, and metabolic dysfunction (138).

Other lifestyle factors may interact with shift work to further increase T2DM risk. For example, night shift nurses who engage in binge drinking showed evidence of circadian misalignment and increased health risks (139). These behavioral patterns suggest that lifestyle interventions should be an important component of T2DM prevention strategies for shift workers. Light therapy has emerged as a promising intervention for mitigating the negative effects of shift work. Studies show that light therapy significantly improves total sleep time and sleep efficiency in shift workers compared to control groups. Specific light parameters appear important—medium illuminance (900–6,000 lux) for longer durations (≥ 1 h) during night shifts was most effective for extending total sleep time, while higher illuminance and increasing dose of light therapy more beneficially impacted sleep efficiency (138). Personalized approaches to light therapy show particular promise. A randomized controlled trial demonstrated that light therapy personalized to an individual's melatonin rhythms produced greater reductions in circadian misalignment compared to standardized approaches. Participants receiving personalized light therapy showed decreased sleepiness and improved sleep quality, suggesting this precision medicine approach could help mitigate shift work disorder symptoms and potentially reduce associated metabolic risks (140).

More sophisticated circadian-informed lighting strategies are also being developed. One study demonstrated that a circadian-informed lighting intervention markedly accelerated circadian adjustment to night shift schedules compared to standard lighting. By administering bright, blue-enriched light and dim, blue-depleted light at specific times calculated to facilitate rapid circadian adaptation, researchers achieved faster adjustment to night shift schedules (141). The heightened T2DM risk in night shift workers is fundamentally linked to circadian disruption, independent of sleep loss, leading to impaired glucose metabolism, insulin sensitivity, and altered hormonal rhythms. This is further exacerbated by suppressed melatonin, adverse gene expression changes, and detrimental behaviors like mistimed eating and sleep deprivation. Moving forward, research should focus on identifying predictive biomarkers for individual susceptibility, optimizing personalized light therapy strategies, and developing comprehensive behavioral and nutritional interventions specifically tailored for shift workers, to ultimately mitigate this health burden.

## 5 Conclusions and future insights

Ultimately, the complex relationship between night shift work and the risk of developing T2DM is influenced by multiple interconnected factors. Key elements, such as circadian disruption, sleep deprivation, and changes in melatonin production, all contribute to the heightened risk faced by individuals working non-traditional hours. While sleep deprivation is a significant factor in the development of T2DM, the specific impacts of night shift work, particularly its effect on biological rhythms, need further exploration. The mechanisms at play are still being unraveled, and more targeted studies are necessary to clarify how these factors interact. Future research should distinguish the effects of night shift work from general sleep deprivation to provide clearer insights. A comprehensive action plan, focused on improving sleep quality, restoring circadian rhythms, and optimizing melatonin production, should be a priority for employers and policymakers. Personalized interventions, considering individual work schedules and genetic predispositions, may hold the key to mitigating T2DM risk in night shift workers. Additionally, the role of diet, exercise, and lifestyle choices in reducing this risk warrants thorough investigation. Longitudinal studies will be essential to monitor the long-term health impacts of night shift work. Collaboration between researchers, employers, and policymakers is crucial to implementing effective workplace policies and fostering awareness among shift workers about the potential health risks. Furthermore, examining the workplace environment and organizational practices can provide additional insight into how night shift work affects health outcomes. Factors such as work-related stress, job satisfaction, and the availability of support systems may also influence the risk of developing T2DM. For instance, workplaces that promote health-conscious behavior through flexible break schedules, healthy meal options, and mental health resources could help mitigate the detrimental effects of night shifts. As such, interventions should not only focus on individual lifestyle changes but also on structural workplace adjustments that prioritize the wellbeing of shift workers. As the field of chronobiology advances, innovative tools and methods may offer further solutions, contributing to better health outcomes for non-traditional workers. Despite the challenges, protecting the physical and mental health of night shift workers remains a vital area of focus for the future of occupational health.
